# Semi-rational engineering membrane binding domain of L-amino acid deaminase from *Proteus vulgaris* for enhanced α-ketoisocaproate

**DOI:** 10.3389/fmicb.2022.1025845

**Published:** 2022-09-30

**Authors:** Yang Song, Rui Wang, Zixuan Zhang, Xinran Liu, Lulu Qi, Xuping Shentu, Xiaoping Yu

**Affiliations:** Zhejiang Provincial Key Laboratory of Biometrology and Inspection and Quarantine, College of Life Science, China Jiliang University, Hangzhou, China

**Keywords:** membrane-binding domain, site-saturation mutagenesis, bioconversion, α-ketoisocaproate, L-amino acid deaminase

## Abstract

α-Keto acids are important raw materials for pharmaceuticals and functional foods, which could be produced from cheap feed stock by whole cell biocatalysts containing L-amino acid deaminases (L-AADs). However, the production capacity is limited by the low activity of L-AADs. The L-AAD mediated redox reaction employs the electron transport chain to transfer electrons from the reduced FADH_2_ to O_2_, implying that the interaction between L-AAD and the cell membrane affects its catalytic activity. To improve the catalytic activity of L-AAD from *Proteus vulgaris*, we redesigned the membrane-bound hydrophobic insertion sequences (INS, residues 325–375) by saturation mutagenesis and high-throughput screening. Mutants D340N and L363N exhibited higher affinity and catalytic efficiency for L-leucine, with half-life 1.62-fold and 1.28-fold longer than that of wild-type L-AAD. D340N catalyzed L-leucine to produce 81.21 g⋅L^–1^ α-ketoisocaproate, with a bioconversion rate of 89.06%, which was 17.57% higher than that of the wild-type. It is predicted that the mutations enhanced the interaction between the protein and the cell membrane.

## Introduction

L-Amino acid deaminase (L-AAD) is a flavin adenine dinucleotide (FAD)-containing protein which can catalyze the deamination of L-amino acids to generate α-keto acids and ammonia (Zhao et al., 2018; Wang et al., 2020). L-AAD has attracted much attention in α-keto acids production because of its widely substrate spectrum (Song et al., 2016). It has been used as a whole-cell biocatalyst to produce α-ketoglutaric acid (Hossain et al., 2014; Liu et al., 2017), α-ketoisovalerate (Li et al., 2017), α-ketoisocaproate (Song et al., 2017), phenylpyruvate (Liu et al., 2022), and α-keto-β-methylvalerate (Yuan et al., 2019). Notably, L-AAD is a membrane-bound enzyme which depends on electron transport chain to transfer electrons from the reduced FADH_2_ to O_2_ without generating H_2_O_2_ (Wu et al., 2020). Therefore, the catalytic performance of L-AAD could be affected by the region of interaction with the cell membrane.

L-Amino acid deaminase binds to the membrane through its N-terminal transmembrane domain (residues 6–29) and a hydrophobic insertion sequence (INS, residues 325–375). The N-terminal transmembrane domain is coded by a twin-arginine translocation (Tat) signal peptide, which exists in the N-terminal of many other transmembrane protein containing redox cofactors. Another hydrophobic insertion domain only exists in this type of L-ADD from *Protues* and *Providencia* (Wu et al., 2021). It is different from amino acid deaminases unbounded to the cell membrane, such as PDH1 from *Pyrococcus horikoshii* (PDB code: 1Y56-B), D-amino acid deaminase from *Rhodotorula toruloides* (PDB code: 1C0P), which use the O_2_ as the direct receptor of electrons to generate H_2_O_2_. The partial three-dimensional (3-D) structure of L-AAD from *Protues vulgaris* has proven that the INS could bound to the cell membrane without the transmembrane domain (Huang et al., 2013). In addition, the enzyme-membrane interaction could be affected by the INS sequence, and the five amino acids mutant in the INS reduces the binding ability and the catalytic activity.

The catalytic ability and substrate selectivity of L-AAD depend on the interaction of the INS domain and cell membrane. When the bacterial cell membrane added, the purified L-AAD increased its catalytic activity by several folds (Kufareva et al., 2014). The deamination mechanism of L-AAD indicated the re-oxidization of FADH_2_ depends on the other membrane protein such as cytochrome *b* as the electron acceptor, which is significantly different from other non-membrane bound deaminase using O_2_ as the acceptor and producing H_2_O_2_ as byproducts (Zhang et al., 2020). Furthermore, the interaction of INS-membrane also improves the catalyst activity and changes the substrate spectrum (Zhang et al., 2020). INS is a flexible domain and the Molecular dynamics (MD) stimulations indicated that INS exhibits dramatic conformational rearrangements in the solution, which could affect the narrow hydrophobic channel for small molecules to come in and out. Mutants in INS also showed changes in substrate spectrum and regulation of the L-AAD substrate preference. Therefore, INS plays a significant role in L-AAD structure and catalysis, but there are few researches in the modification of INS to improve the catalytic properties (Asano and Yasukawa, 2019).

α-Ketoisocaproate is the precursor of the branch-chained amino acid L-leucine, which could be used as the nitrogen-free substitutes for leucine to provide patients with their daily requirement of L-leucine. The α-ketoisocaproate combined with a low-nitrogen diet could effectively decrease the accumulation of urea and other metabolic nitrogenous waste from dietary protein for patients with the chronic kidney and hepatic disorder (Song et al., 2017). So, the α-ketoisocaproate is an important chemical compound in pharmaceutical and nutraceutical industries. In our previous study, L-AAD from *P. vulgaris* was expressed in *Escherichia coli*, displaying catalytic activity toward L-leucine (Song et al., 2015, 2017). By optimization of RBS and plasmid copy number, the expression amount of enzyme and the production of α-ketoisocaproate were improved. However, activity and thermal stability limited the ability of L-ADD to catalyze the production of α-ketoisocaproic acid. The total bioconversion reaction needed 24 h or longer, which means the low productivity in the industrial process. Furthermore, the low thermal stability (the half-lives of this enzyme at 37°C was only 8 h) of wild-type L-AADs renders the enzyme unsuitable for long term reactions and prevents the increase of α-keto acid production (Supplementary Figure 1). Therefore, it is essential to improve the activity and thermostability at the same time, which means higher productivity in the industrial production. To this end, INS were designed in this study to enhance the activity and thermal stability of L-AAD. Mutants with high activity and thermostability were screened for α-ketoisocaproate synthesis from L-leucine by saturation mutagenesis and high-throughput screening. Overall, semi-rational engineering strategy to improve catalytic properties by modifying membrane-bound domain though site-saturation mutagenesis may be useful for the directed evolution of the other biocatalysts.

## Materials and methods

### Bacterial strains, plasmids, and materials

The plasmid pET28a-*lad* with wild-type L-AAD gene and the recombinant strain *E. coli* BL21-lad were constructed in a previous study (Song et al., 2015). The restriction endonucleases, PCR reagents, blunting kit used for saturation mutagenesis were purchased from Takara (Dalian, China). The standard of α-ketoisocaproate was purchased from Sig-ma-Aldrich (St. Louis, MO, USA), and other reagents were purchased from Sangon Biotech Co. (Shanghai, China).

### Library creation and primary screening for better catalytic and temperature characteristics

The recombinant plasmids were constructed through whole-plasmid PCR using the NNK codon substitution at target sites. N represents A, T, C, or G; K represent G or T as shown in Table 1. The PCR template used was pET28a-*lad*, and PCR conditions were 98°C for 2 min; 25 cycles of 98°C for 10 s, 55°C for 5 s, 72°C for 6 min; ended with 10 min of 72°C. The PCR product was then digested with *Dpn*I to remove the parent plasmid and later purified with a PCR purification kit. The purification product was blunted and phosphorylated by Blunting kit, and ligated by T4 ligase. The recombinant plasmid was transformed into *E. coli* BL21 (DE3) and selected using kanamycin at 50 μg ml^–1^ on an LB agar plate. The selected colonies were cultured at 37°C overnight in LB broth in 96-well deep plates. These cultures were then inoculated at a ratio of 1:100 into two 96-well deep plates with fresh TB broth and cultured at 37°C. Upon reaching an OD_600_ of approximately 0.6, 0.4 mM isopropyl-β-d-thio-galactoside (IPTG) was added to the wells. The cells were harvested after induced for 4 h by spinning the plates at 4,000 rpm and 4°C for 10 min. Then the substrate 100 mM L-leucine was added into one plate to measure bioactivity and temperature characteristic as follows.

**TABLE 1:** The sequences of primers.

Primer	Sequences (5′−3′)
F326-F	**NNK**ACTTATGGCTATAAATATCTGCCAT
F326-D	GGATTCTTTCACTACTGGCGC
D340-F	**NNK** TTCCCTGTGCATATTTCTTTAAATGAAC
D340-D	AGGTAATGCTAATAATGGCAGATATTTATAGC
L347-F	**NNK**AATGAACAATTAATCAATTCATTTATGCAATCAAC
L347-D	AGAAATATGCACAGGGAAATCAGGTAA
I352-F	**NNK**AATTCATTTATGCAATCAACGCATTG
I352-D	TAATTGTTCATTTAAAGAAATATGCACAGGG
F355-F	**NNK**ATGCAATCAACGCATTGGAAC
F355-D	TGAATTGATTAATTGTTCATTTAAAGAAATATGCA
L363-F	**NNK**GATGAAGTTTCTCCGTTTGAGCAATTCAGAAATATG
L363-D	GTTCCAATGCGTTGATTGCATAAATGAATTGA

NNK represents the mutant codon used in this study.

The variants were primarily screened by comparison of initial catalytic activity of the whole-cells. The α-ketoisocaproate were evaluated by the chromogenic reaction of 2, 4-dinitrophenylhydrazine (DNP). The initial biocatalytic activity was measured using 100 mM L-leucine as substrate (20 mm phosphate buffer, pH 7.5) at 37°C for 30 min with whole-cells in the 96-well deep plates. The reaction was stopped through centrifugation at 10,000 rpm for 10 min. Afterward, 50 μl of the supernatant was mixed with 100 μl 20 mm DNP at 25°C for 5 min, followed by the addition of 200 μl 1.5 M NaOH solution to 30 μl of the supernatant mixture. Absorbance was then measured at 520 nm. The relative activity change was calculated by setting the wild-type L-AAD as the standard (0%). The temperature characteristics were measured by incubating at 37°C for 480 min in phosphate buffer (20 mM, pH 7.5) in 96-well deep plates for the primary screening for the thermostability.

### Protein expression and purification

The recombinant *E. coli* BL21 (DE3) was inoculated into 20 ml LB broth with 50 μg ml^–1^ kanamycin and cultured overnight at 37°C and 220 rpm. This seed culture was then inoculated into TB broth to a final concentration of 1% (v/v) to induce the expression of the protein. IPTG (final concentration, 0.4 mM) was added upon reaching OD_600_ of 0.6. Induction for the recombinant strains using IPTG at 37°C was done for 4 h. The cells were then collected by centrifugation at 8,000 × *g* for 10 min. The protein was then purified following the same method as in a previous study (Hou et al., 2015) except the use Triton X-100 as the detergent. HisTrap™ FF Ni affinity column (1 ml, GE Healthcare) was used to purify the protein with AKTA Explorer and the protein concentration was determined using a BCA protein assay kit (TianGen, Beijing, China).

### Biocatalytic property assays

The enzyme activity was determined by measuring the production of α-ketoisocaproate using L-leucine as the substrate. A volume of 1 ml L-leucine (100 mM) and final cell density of 0.8 g L^–1^ whole-cell were mixed, then incubated at 37°C for 30 min, 200 μl 20% trichloroacetic acid was then added to stop the reaction. The mixture was subjected to centrifugation at 8,000 × *g* for 10 min. The supernatant was then analyzed using HPLC to determine the α-ketoisocaproate concentration. HPLC conditions used were the same as in a previous study (Song et al., 2015). One unit (U) of activity was defined as the amount of enzyme producing 1 μmol α-ketoisocaproate under these conditions.

The optimal stability of L-AAD was measured within the range of 20–50°C, and the optimal pH of L-AAD was determined within the range of pH 5.5–9.5. The inactivation half-life of L-AAD was determined at 37°C in phosphate buffer (pH 7.5, 20 mM) for 24 h. The pH stability of L-AAD was measured at pH ranging from 5.5 to 9.5 at 37°C for 24 h. After incubation, the enzyme activity was measured at pH 7.5 and 37°C. The activity of wild-type L-AAD incubated at above conditions was set as the standard (100%).

The kinetic parameters (*K*_m_, *V*_max_, *K*_cat_, and *K*_cat/_*K*_m_) were measured in phosphate buffer (pH 7.5, 20 mM) at 37°C with the substrate concentrations ranging from 0 mM to 175 mM. The *K*_m_ and *V*_max_ values were calculated by fitting the activity at different substrate concentration to Michaelis–Menten equation by Origin 8.5. The *K*_cat_ was calculated by dividing the *V*_max_ by enzyme molar concentration.

### Whole-cell bioconversion of L-leucine to α-ketoisocaproate by mutants

The harvested cells were washed twice and suspended by in a 100 mM L-leucine solution (pH 7.5) with final cell density of 0.8 g L^–1^ in 250-ml flasks. The reaction was performed at optimal temperature, 220 rpm. Afterward, sterile L-leucine powder was added into the reaction solution with a feed rate of 6.55 g L^–1^ h^–1^ for the first 12 h (13.1 g L^–1^ L-leucine was added every 2 h, total of 6 additions). Samples of the mixture were obtained every 4 h to measure the α-ketoisocaproate concentrations following procedures used in a previous study (Song et al., 2015).

### Molecular dynamics simulation

The crystal structure of L-AAD was obtained from Protein Data Bank (5hxw) and prepared using Discovery Studio 4.1 (Accelrys, San Diego, CA, USA). The protonation states of charged residues were identified using the H^++^ program (Gordon et al., 2005), and the protein was solvated into a box with 10 Å H_2_O (Jorgensen et al., 1983), with Na^+^ or Cl^–^ ions to neutralize the charges using Amber 16 (Case et al., 2005). The Amber99 SB and general Amber force field (gaff) force field (Maier et al., 2015) were used to assign protein and its corresponding FAD. The model was first subjected to a steep descent energy minimization, and then heated from 0 K to 300 K with fixed heavy and Cα atoms. Then, a 100 ps of isothermal-isobaric ensemble (NPT) MD simulation was used to keep the temperature and pressure constant (ρ = 1.0 g cm^–3^, *T* = 300 K, *P* = 1.0 atm). Finally, a 10-ns MD simulation was performed on the whole system at 300 K, treated with periodic boundary condition. The root mean square deviation (RMSD) and root mean square fluctuation (RMSF) values were calculated with ptraj program in Amber 16.

## Results

### The selection of the key residues based on the structure analysis

**FIGURE 1:**
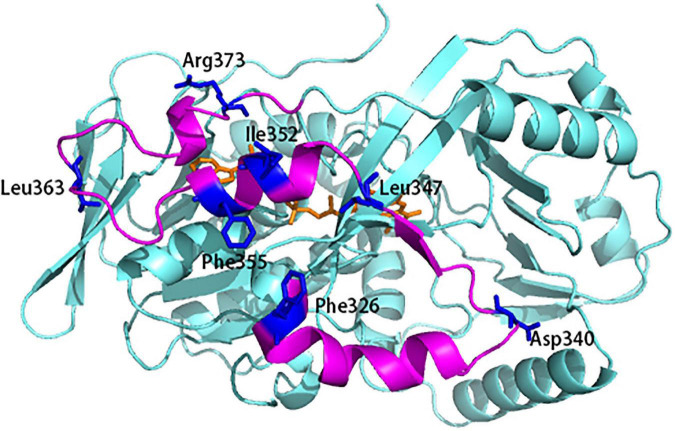
The flexibility and potential residues chosen for site-saturation mutagenesis. The 3D model was based on the X-ray crystallographic structure of L-amino acid deaminase (L-AAD) (PDB: 5hxw). Mutant sites were shown in blue.

Since INS plays a critical role in the structural stability and bioactivity, we first identified the key residues in INS which are potentially involved in enhancing the bioactivity and thermostability of L-AAD. The INS contain 3 α helix and 1 β sheet, linked by two big loop regions. In general, hydrophobic residues located on α-helices and β-sheets (residues 325–226, 344–356, and 368–375) affect enzyme activity, so a sequence alignment of the INS was conducted to find the conversed hydrophobic residues in the L-AAD from *P. vulgaris* (Supplementary Figure 2). Eleven residues (F326, L333, L335, L336, I345, L347, L351, I352, F355, P368, and F369) were identified (colored in purple in Supplementary Figure 2). Among them, eight residues (F326, L333, I345, L347, L351, I352, F355, and P368) were located on the surface of the domain, which may be related to the interaction with the cell membrane. So these eight residues were mutated to alanine to test the effect on catalytic activity (Supplementary Figure 3A). The results showed that mutants F326A, L347A, I352A, and F355A could significantly reduce the activity of L-AAD by more than 73.25%. In addition, the loops often affect stability of enzyme due to the structural instability. To this end, conserved residues in the two big loops of the INS (residues 337–343 and residues 357–367) were identified to explore the possibility of improving stability (D340, F341, P342, V343, T359, W361, L363, D364, E365, and S367) (colored in blue in Supplementary Figure 2). The alanine scanning analysis (Supplementary Table 1) indicated that the five residues (D340, F341, V343, L363, and E365) could improve the thermostability (ΔG > 0). Likewise, replacing these residues with alanine to test for changes in thermal stability showed that D340 and L363 significantly reduced the thermal stability of L-AAD (Supplementary Figure 3B). Taken together, the surface exposed hydrophobic residues F326, L347, I352, F355 on the α-helix and β-sheet and D340, L363 at the ends of the two big loop regions were selected to improve the interaction of L-AAD with the membrane. The target residues were colored blue in the Figure 1 to show the spatial location.

### Site-directed mutagenesis of L-amino acid deaminase for higher catalytic activity and thermostability

After selecting seven residues with potential ability to improve catalytic activity and thermostability, site specific mutagenesis was done through the replacement of these seven codons with NNK (Table 1). Subsequently, *E. coli* carrying the mutants were induced to express in 96-well plates, and their biocatalytic activity and thermostability were measured based on whole-cell catalysis (Supplementary Figure 4).

**FIGURE 2:**
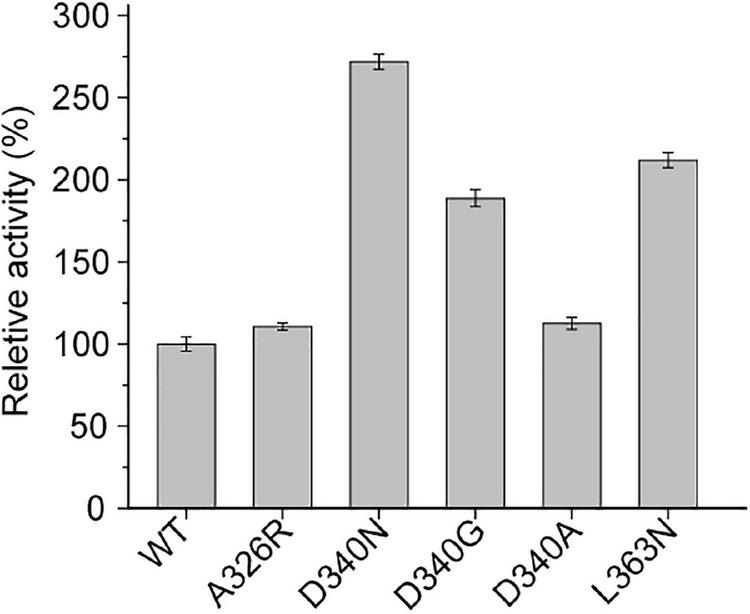
The secondary screening of wild-type (WT) and mutants at 50°C.

Among them, mutants at sites D340 and L363 on the loop of INS showed higher activity than the wild-type L-AAD at 37°C. Interestingly, the thermal stability of the D340 mutant was significantly improved over the wild type, while retaining biocatalytic activity comparable to the wild type. The positive mutants were sequenced and identified as D340N, D340G, D340A, and L363N. Afterward, a secondary screening step was performed, by incubating cells expressing the mutants at 50°C for 1 h to measure the thermostability. Then the biocatalyst activity was determined at 37°C. As shown in Figure 2, the catalytic activity of mutant D340N increased by 149.2% after heat treatment, and the catalytic activity of mutant L363N increased by 117.56%. In order to verify whether the enzyme’s activity could be improved further, an iterative saturation mutagenesis of D340 and L363 were performed. However, a dramatic decrease in activity and thermostability was observed as shown in Supplementary Figure 5. These results confirm the importance of these two amino acids in maintaining the protein’s activity and thermo-stability, but do not have additive effects.

### The catalytic properties of the wild-type L-amino acid deaminase and mutants

**FIGURE 3:**
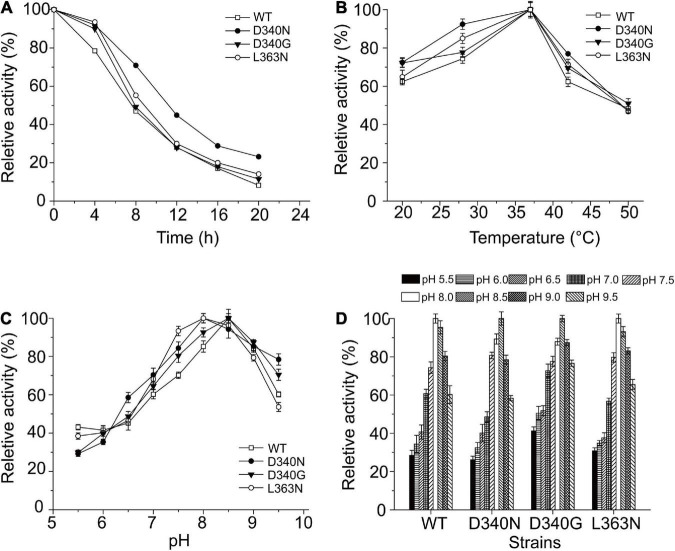
The influence of reaction temperature and pH on the wild type and mutants with L-leucine as substrate. **(A)** Thermal profiles of wild-type (WT) and mutants L-amino acid deaminase (L-AAD). **(B)** The optimal temperature of L-AAD and its mutants. **(C)** The optimal pH of L-AAD and its mutants. **(D)** The pH stability of L-AAD and its mutants. The maximal value of catalytic activity was defined as 100%. Error bars represent SD.

Figures 3A,B shows the observed thermostability of the wild-type L-AAD and the mutants of L-AAD. The half-lives (t1/2) at 37°C of mutants D340N and L363N were 11.4 and 8.9 h, which were 1.62- and 1.28-fold that of L-AAD (7 h), respectively. However, the temperature optima (T_opt_) of D340N and L363N, at 35°C, were the same as that of the wild-type L-AAD (Figure 3B). The effect of pH on the enzyme activity and stability is shown in Figures 3C,D. The optimal pH of wild-type L-AAD and D340G was found to be 8.5, while that of mutants D340N and L363N decreased to 8.0. The stable pH range of the mutants D340N and L363N was the same as that of L-AAD (pH 7.5–9.0); their bioactivity decreased sharply at conditions below pH 7.0, whereas the D340G mutant showed higher activity under pH 7.0. These results suggested that the mutations at D340 and L363 were critical for the improvement of L-AAD activity and thermostability.

### Kinetic characterization of L-amino acid deaminase and mutants

The *K*_m_ and *V*_max_ values of the wild-type and mutants D340N, D340G, and L363N were measured at the optimal temperature and pH (Table 2). The *V*_max_ values of the three mutants were found to be higher than that of the wild-type L-AAD, among the mutants, the D340N showed the highest *V*_max_ (2.67 μmol⋅min^–1^⋅mg^–1^), increased 64.8% than the V_max_ of wild-type. In addition, the *K*_m_ of D340N decreased to the 9.87 mmol⋅L^–1^, which is lower than the wild-type and other mutants. The overall catalytic efficiency *K*_cat_*_/_K*_m_ values of the mutants D340N, D340G, and L363N were 235.39, 105.74, and 107.34 s^–^1⋅mol^–1^, respectively. This indicated that these mutants, particularly D340N, had a higher affinity and catalytic efficiency toward the substrate L-leucine than wild-type L-AAD.

**TABLE 2:** Comparison of properties of the wild-type (WT) and mutants (D340N, D340G, and L363N). *K*_m_ and *K*_cat_ values were calculated by Michaelis–Menten equation.

Mutant	*K*_m_ (mmol⋅L^–1^)	*K*_cat_ (s^–1^)	*K*_cat_/*K*_m_ (s^–1^⋅mol^–1^)	*V*_max_ (μ mol⋅min^–1^⋅mg^–1^)
WT	17.71	1.41	79.55	1.62
D340N	9.87	2.32	235.39	2.67
D340G	16.01	1.69	105.74	1.94
L363N	15.87	1.70	107.34	1.92

### Influence of mutants on fed-batch biotransformation process for α-ketoisocaproate production

**FIGURE 4:**
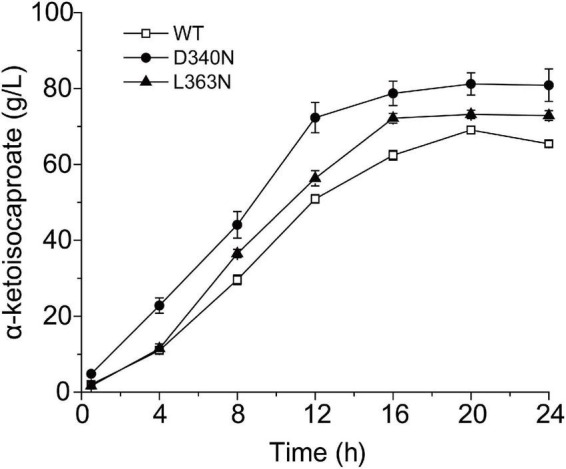
The time profiles of α-ketoisocaproate synthesis by the wild-type (WT) L-amino acid deaminase (L-AAD) and mutants with L-leucine as substrate.

Finally, the whole cell biotransformation of L-leucine to α-ketoisocaproate using the thermostable mutants D340N and L363N was measured (Figure 4). It was observed that the α-ketoisocaproate concentrations using D340N and L363N reached 81.21 and 73.21 g⋅L^–1^, with bioconversion yields of 89.06 and 80.28%, respectively (Figure 4). The production capability of D340N and L363N increased by 17.57 and 5.99%, respectively, compared to that of the wild-type L-AAD. Therefore, the enzyme’s catalytic properties were correlated with the bioconversion efficiency and production of α-ketoisocaproate.

## Discussion

The bioconversion of L-amino acids to α-keto acids by L-AAD is the most effective approach to prepare α-keto acids, which is a potential industrial process to replace chemical synthesis. The bioconversion efficiency could be enhanced by improvement of enzyme kinetic properties and thermal stability by protein engineering methods. In this study, the activity and thermal stability of L-AAD from *P. vulgaris* was tackled by a semi-rational design of INS domain. In addition, the application of the best mutants in bioconversion of L-leucine to α-ketoisocaproate was determined and α-ketoisocaproate production increased by 17.57%. The engineered L-AAD has important implications for the production of α-keto acids by whole-cell bioconversion.

**FIGURE 5:**
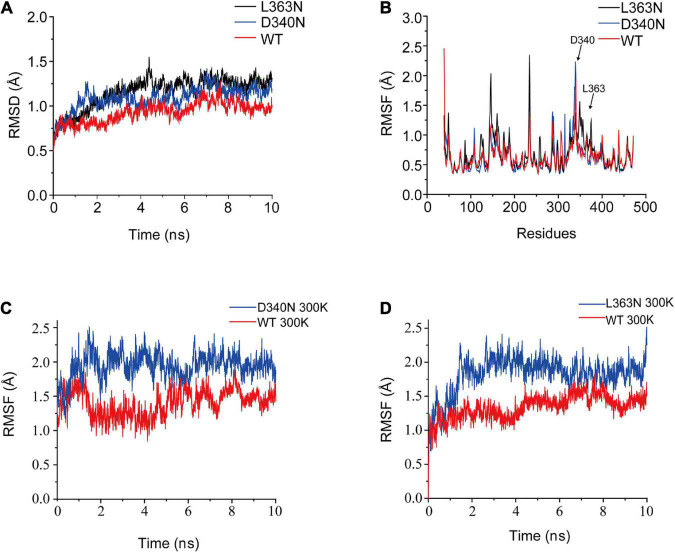
The RMSD and RMSF values for L-amino acid deaminase (L-AAD) and its mutants. The RMSD **(A)** and RMSF **(B)** values for the whole protein L-AAD and its mutants. **(C)** The RMSD of loop D340 for the wild-type L-AAD and D340N. **(D)** The RMSD of loop L363 for the wild-type L-AAD and L363N.

To understand the mechanism underlying improved thermostability, we conducted MD simulations at 300 K to analyze the structural changes in the mutants (Figure 5). The RMSD values of the backbone atoms of L-AAD and mutants D340N and L363N were shown in Figure 5. The RMSD and RMSF values of D340N and L363N were found to be higher than that of wild-type, indicating that the structures of the mutants were more unstable than that of the wild-type. The RMSD values of the loops of both D340N and L363N also showed an increase in fluctuation at 300 K, compared with that of the wild-type L-AAD, which contradicted observations of increased thermostability for these two mutants. However, it is important to note that the simulation did not account for the membrane as did experimental system. Therefore, it was presumed that the membrane played a critical role in the thermostability of the protein. That the membrane-bound form of an enzyme is more thermostable than the soluble enzyme has been shown with the membrane-bound monoamine oxidase from pig-liver mitochondria, the membrane-bound oxalate oxidase, and the membrane-bound hydrogenase (Oreland and Ekstedt, 1972; Goyal et al., 1999; McTernan et al., 2014).

**FIGURE 6:**
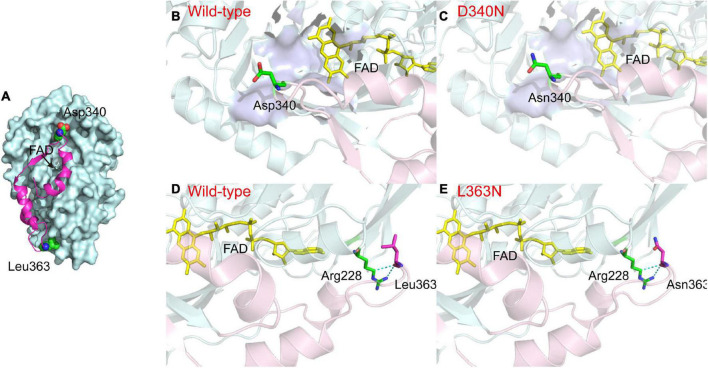
Comparison between the structures of L-amino acid deaminase (L-AAD) and mutants. **(A)** The overall structure of L-AAD. The INS region was shown as the magentas cartoon. **(B–E)** The difference of side chain in L-AAD and mutants. The INS was shown as the cartoon by light pink and the rest of the protein was colored by light blue. The active sites of L-AAD were mod-eled based on the crystallographic structures (PDB: 5hxw, the active center was shown as the surface in purple blue). The mutant residues were represented as sticks and H-bond was shown as the dotted lines.

The L-AAD is a membrane-bound protein with an N-terminal transmembrane region (located between amino acid residues 1–29) and a hydrophobic insertion sequence on the surface (INS, located between amino acid residues 321–375), for binding and interaction with the membrane. The INS region is composed of three α-helices and one β-strand. The central region of the INS was composed of several hydrophobic amino acid residues, which could stabilize the L-AAD structure on the membrane and affect its catalytic activity (Xiong et al., 2021). The critical sites D340 and L363 were located on both ends of the loop in the INS region, indicating that the D340 and L363 may have significant functions in cell membrane interaction (Figure 6A). The notable difference in the charges of the side chains of aspartic acid, leucine, and asparagine is shown in Figures 6B–D. The isoelectric point for most strains was pH 3.0–4.0, resulting in a negative charge of the outer membrane in a pH 7.5 solution (Li and McLandsborough, 1999). In D340N, a negative amino acid (aspartic acid) was replaced by a neutral amino acid (asparagine), which might have decreased the repulsive interaction with the negative membrane. For the L363N mutant, a hydrophobic residue (leucine) was replaced by the asparagine with more hydrophile groups, which may increase the interaction with the outer membrane surface (Figure 6E). In addition, the H-bond distances (determined using Discovery Studio 4.1) around residue L363 of the mutant L363N increased from 2.94 and 2.91 Å to 2.95 and 2.95 Å, respectively, indicating a decrease in the interaction of L363 with R228. Therefore, we hypothesize that the enhanced activity and thermostability of the D340N and L363N mutants may be attributed to the increase in interaction between the enzyme and the cell membrane.

In conclusion, the activity of L-AAD was remarkably improved via the improvement of interaction of enzyme and membrane. Another distinguishing characteristic of the mutants was that their higher thermostability compared with the wild-type, which is rare in engineered proteins that both properties improved simultaneously. This study revealed the importance of protein and membrane interaction in enzyme catalysis and provided a viable strategy to enhance product yield.

## Data availability statement

The original contributions presented in the study are included in the article/Supplementary material, further inquiries can be directed to the corresponding authors.

## Author contributions

YS: conceptualization, resources, writing—original draft preparation, and funding acquisition. XS: methodology and project administration. ZZ: software and data curation. RW: validation. YS, XL, and LQ: investigation. XY: writing—review and editing and supervision. All authors contributed to the article and approved the submitted version.

## References

[B1] Asano Y. Yasukawa K. (2019). Identification and development of amino acid oxidases. *Curr. Opin. Chem. Biol.* 49 76–83. 10.1016/j.cbpa.2018.10.020 30448541

[B2] Case D. A. Cheatham T. E. Darden T. Gohlke H. Luo R. Merz K. M. (2005). The Amber biomolecular simulation programs. *J. Comput. Chem.* 26 1668–1688. 10.1002/jcc.20290 16200636 PMC1989667

[B3] Gordon J. C. Myers J. B. Folta T. Shoja V. Heath L. S. Onufriev A. (2005). H++: A server for estimating pK as and adding missing hydrogens to macromolecules. *Nucleic Acids Res.* 33 368–371. 10.1093/nar/gki464 15980491 PMC1160225

[B4] Goyal L. Thakur M. Pundir C. S. (1999). Purification and properties of a membrane bound oxalate oxidase from *Amaranthus* leaves. *Plant Sci.* 142 21–28. 10.1016/S0168-9452(98)00251-9

[B5] Hossain G. S. Li J. Shin H.-d. Chen R. R. Du G. Liu L. (2014). Bioconversion of L-glutamic acid to α-ketoglutaric acid by an immobilized whole-cell biocatalyst expressing L-amino acid deaminase from *Proteus mirabilis*. *J. Biotechnol.* 169 112–120. 10.1016/j.jbiotec.2013.10.026 24172254

[B6] Hou Y. Hossain G. S. Li J. Shin H.-d. Liu L. Du G. (2015). Production of phenylpyruvic acid from L-phenylalanine using an L-amino acid deaminase from *Proteus mirabilis*: comparison of enzymatic and whole-cell biotransformation approaches. *Appl. Microbiol. Biotechnol.* 99 8391–8402. 10.1007/s00253-015-6757-0 26109004

[B7] Huang H. K. Taneva S. G. Lee J. Silva L. P. Schriemer D. C. Cornell R. B. (2013). The membrane-binding domain of an amphitropic enzyme suppresses catalysis by contact with an amphipathic helix flanking its active site. *J. Mol. Biol.* 425 1546–1564. 10.1016/j.jmb.2012.12.003 23238251

[B8] Jorgensen W. L. Chandrasekhar J. Madura J. D. Impey R. W. Klein M. L. (1983). Comparison of simple potential functions for simulating liquid water. *J. Chem. Phys.* 79 926–935. 10.1063/1.445869

[B9] Kufareva I. Lenoir M. Dancea F. Sridhar P. Raush E. Bissig C. (2014). Discovery of novel membrane binding structures and functions. *Biochem. Cell Biol.* 92 555–563. 10.1139/bcb-2014-0074 25394204 PMC4267288

[B10] Li J. McLandsborough L. (1999). The effects of the surface charge and hydrophobicity of *Escherichia coli* on its adhesion to beef muscle. *Int. J. Food Microbiol.* 53 185–193. 10.1016/s0168-1605(99)00159-2 10634709

[B11] Li R. Sakir H. G. Li J. Shin H.-d. Du G. Chen J. (2017). Rational molecular engineering of L-amino acid deaminase for production of α-ketoisovaleric acid from L-valine by *Escherichia coli*. *RSC Adv.* 7 6615–6621. 10.1039/C6RA26972A

[B12] Liu J. Liu J. Yang B. Gao C. Song W. Hu G. (2022). Production of phenylpyruvic acid by engineered L-amino acid deaminase from *Proteus mirabilis*. *Biotechnol. Lett.* 44 635–642. 10.1007/s10529-022-03245-y 35429303

[B13] Liu Q. Ma X. Cheng H. Xu N. Liu J. Ma Y. (2017). Co-expression of L-glutamate oxidase and catalase in *Escherichia coli* to produce α-ketoglutaric acid by whole-cell biocatalyst. *Biotechnol. Lett.* 39 913–919. 10.1007/s10529-017-2314-5 28251390

[B14] Maier J. A. Martinez C. Kasavajhala K. Wickstrom L. Hauser K. E. Simmerling C. (2015). ff14SB: improving the accuracy of protein side chain and backbone parameters from ff99SB. *J. Chem. Theory Comput.* 11 3696–3713. 10.1021/acs.jctc.5b00255 26574453 PMC4821407

[B15] McTernan P. M. Chandrayan S. K. Wu C.-H. Vaccaro B. J. Lancaster W. A. Yang Q. (2014). Intact functional fourteen-subunit respiratory membrane-bound [NiFe]-hydrogenase complex of the hyperthermophilic archaeon *Pyrococcus furiosus*. *J. Biol. Chem.* 289 19364–19372. 10.1074/jbc.M114.567255 24860091 PMC4094048

[B16] Oreland L. Ekstedt B. (1972). Soluble and membrane-bound pig liver mitochondrial monoamine oxidase: Thermostability, tryptic digestability and kinetic properties. *Biochem. Pharmacol.* 21 2479–2488. 10.1016/0006-2952(72)90419-4 4646794

[B17] Song Y. Li J. Shin H. d. Du G. Liu L. Chen J. (2015). One-step biosynthesis of α-ketoisocaproate from L-leucine by an *Escherichia coli* whole-cell biocatalyst expressing an L-amino acid deaminase from *Proteus vulgaris*. *Sci. Rep.* 5:12614. 10.1038/srep12614 26217895 PMC4517468

[B18] Song Y. Li J. Shin H. Liu L. Du G. Chen J. (2016). Biotechnological production of alpha-keto acids: Current status and perspectives. *Bioresour. Technol.* 219 716–724. 10.1016/j.biortech.2016.08.015 27575335

[B19] Song Y. Li J. Shin H.-d. Liu L. Du G. Chen J. (2017). Tuning the transcription and translation of L-amino acid deaminase in *Escherichia coli* improves α-ketoisocaproate production from L-leucine. *PLoS One* 12:e0179229. 10.1371/journal.pone.0179229 28662040 PMC5491005

[B20] Wang J. Song W. Wu J. Liu J. Chen X. Liu L. (2020). Efficient production of phenylpropionic acids by an amino-group-transformation biocatalytic cascade. *Biotechnol. Bioeng.* 117 614–625. 10.1002/bit.27241 31803933

[B21] Wu L. Guo X. Wu G. Liu P. Liu Z. (2020). Efficient enzymatic synthesis of α-keto acids by redesigned substrate-binding pocket of the L-amino acid deaminase. *Enzyme Microb. Technol.* 132:109393. 10.1016/j.enzmictec.2019.109393 31731950

[B22] Wu Y. Zhang S. Song W. Liu J. Chen X. Hu G. (2021). Enhanced catalytic efficiency of L-amino acid deaminase achieved by a shorter hydride transfer distance. *ChemCatChem* 13 4557–4566.

[B23] Xiong T. Bai Y. Fan T. P. Zheng X. Cai Y. (2021). Biosynthesis of phenylpyruvic acid from L-phenylalanine using chromosomally engineered *Escherichia coli*. *Biotechnol. Appl. Biochem.* 1–8. 10.1002/bab.2256 34554609

[B24] Yuan Y. Song W. Liu J. Chen X. Luo Q. Liu L. (2019). Production of α-ketoisocaproate and α-keto-β-methylvalerate by engineered L-amino acid deaminase. *ChemCatChem* 11 2464–2472. 10.1002/cctc.201900259

[B25] Zhang D. P. Jing X. R. Fan A. W. Liu H. Nie Y. Xu Y. (2020). Active expression of membrane-bound L-amino acid deaminase from *Proteus mirabilis* in recombinant *Escherichia coli* by fusion with maltose-binding protein for enhanced catalytic performance. *Catalysts* 10:215. 10.3390/catal10020215

[B26] Zhao W. Ding H. Lv C. Hu S. Huang J. Zheng X. (2018). Two-step biocatalytic reaction using recombinant *Escherichia coli* cells for efficient production of phenyllactic acid from L-phenylalanine. *Process Biochem.* 64 31–37. 10.1016/j.procbio.2017.09.019

