# Supplementary material for: Semi-rational engineering membrane binding domain of L-amino acid deaminase from Proteus vulgaris for enhanced α-ketoisocaproate
Source: Front Microbiol. 2022 Sep 30;13:1025845. doi: 10.3389/fmicb.2022.1025845 (PMC9561763; doi:10.3389/fmicb.2022.1025845)

**Semi-rational engineering membrane binding domain of L-amino acid deaminase from Proteus vulgaris for enhanced α-ketoisocaproate**

Yang Song, Rui Wang, Zixuan Zhang, Xinran Liu, Lulu Qi, Xuping Shentu*, Xiaoping Yu*

Zhejiang Provincial Key Laboratory of Biometrology and Inspection and Quarantine, College of Life Science, China Jiliang University, Hangzhou 310018, China

*Correspondence:

Prof. Xuping Shentu; E-mail address: stxp@cjlu.edu.cn; Tel: +86-571-86876237

Prof. Xiaoping Yu; E-mail address: yxp@cjlu.edu.cn; Tel: +86-571-86836006

**Table 1** The mutation energy (ΔG) was determined by the alanine scanning in Discovery Studio 4.5.

| Residues | Mutation energy (ΔG) |
| --- | --- |
| D340 | 0.65 |
| F341 | 1.55 |
| P342 | -0.6 |
| V343 | 1.62 |
| T359 | 0 |
| W361 | -0.3 |
| L363 | 0.25 |
| D364 | -0.31 |
| E365 | 1.88 |
| S367 | -0.32 |

**Figure Caption**

**Figure S1** The thermostability of L-AAD under 37 37°C. The experiment was conducted under the phosphate buffer (pH 7.5)

**Figure S2** The sequence alignments of INS and mutant residues.

**Figure S3** The biocatalyst activity and thermostability of residues mutant to alanine. (A) The relative activity of residues mutant to alanine. (B) The thermostability of residues mutant to alanine. The biocatalyst activity and thermostability of wild-type were defined as 100%. The measurement was repeated for three times and the SD value was used as the error bar.

**Figure S4** Primary screening of the site-mutagenesis library. The horizontal ordinate showed the catalytical activity and vertical coordinate represent the thermostability. The wild-type was used as the reference and showed as a red point.

**Figure S5** The secondary screening of iterative saturation mutagenesis. The horizontal ordinate showed the catalytical activity and vertical coordinate represent the thermostability. The wild-type was used as the reference and showed as a red point.

Figure S1


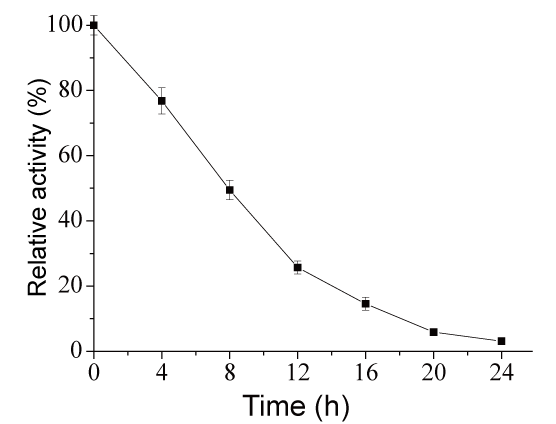


The results indicated that the relative catalytic activity decrease sharply during 12 h, and the half-lives t_(1/2)_ of this enzyme at 37 °C was only 8 h, which is much shorter than the total bioconversion time 24 h. Besides, the production curve of α-ketoisocaperate by L-amino acid deaminase showed that the production of α-ketoisocaperate increased slowly after reaction for 8 h. So the thermostability should be improved to improve the α-ketoisocaperate production.

Figure S2


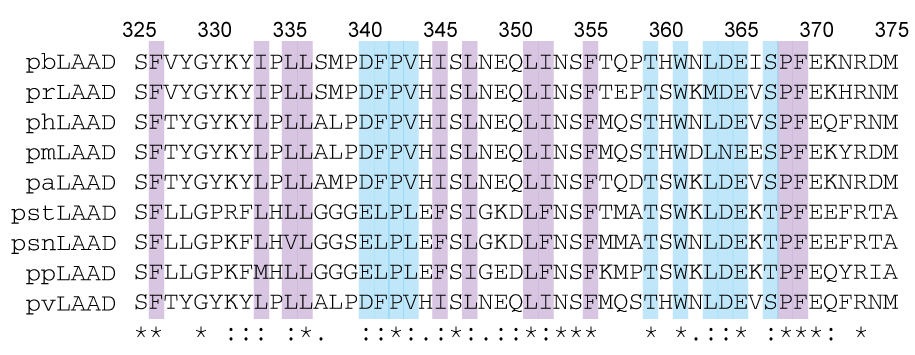


The sequences L-amino acid deaminase from Providencia burhodogranariea DSM 19968 (pbLAAD, GeneBank accession number EKT62719), Providencia rettgeri Dmel1 (prLAAD, GeneBank accession number EKT57949), Proteus hauseri ZMd44 (phLAAD, GeneBank accession number EST56802), roteus mirabilis (pmLAAD, GeneBank accession number ACD36582), Providencia alcalifaciens Dmel2 (paLAAD, GeneBank accession number EKT66496), *Providencia stuartii* MRSN 2154 (pstLAAD, Genebank AFH94393.1), *Providencia sneebia* DSM 19967 (psnLAAD, Genebank EKT60322.1), *Proteus penneri* (ppLAAD, GenBank SUC02995.1), Proteus vulgaris (pvLAAD, GeneBank accession number BAA90864) were compared using ClustalW program. The identified hydrophobic residues were color purple in the 3 α helix and 1 β sheet, and the residues colored in blue located in the loops.

Figure S3


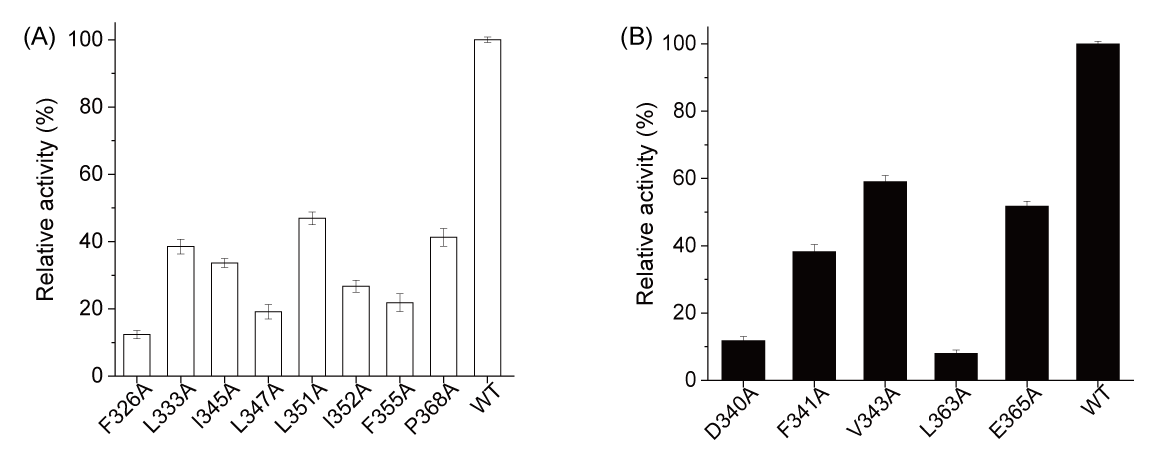


Figure S4


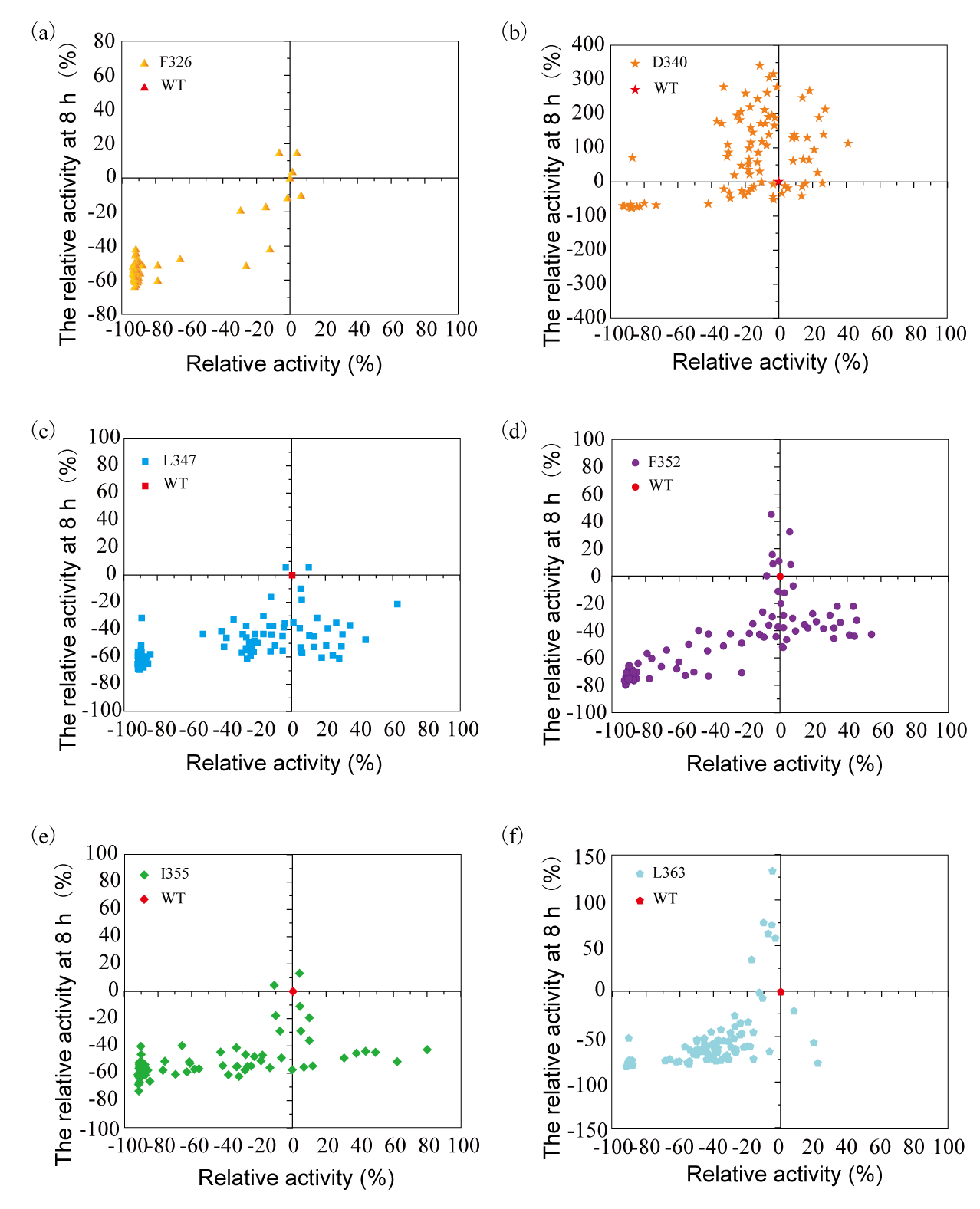


Figure S5


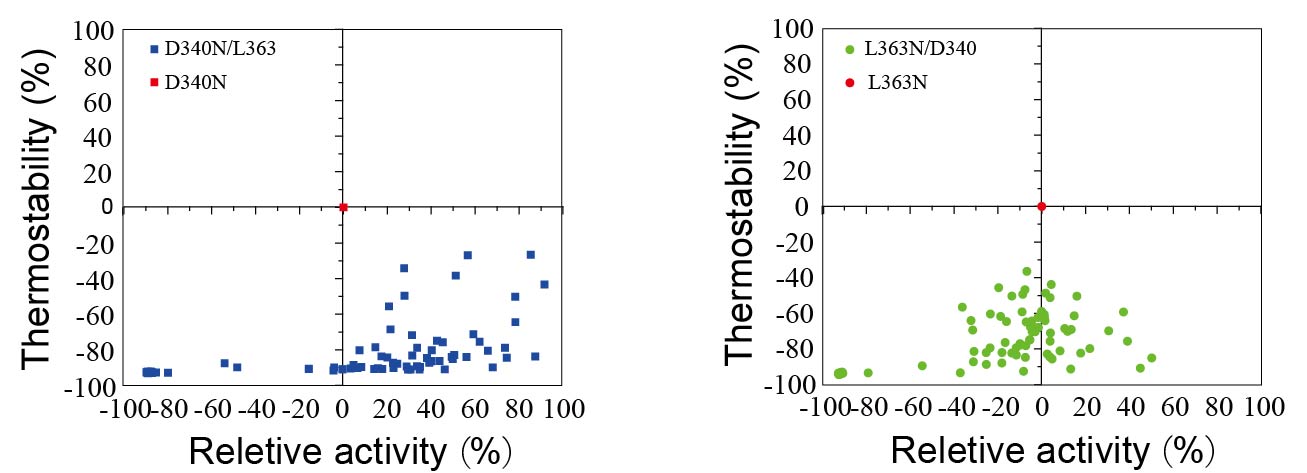

Supplement: Supplementary file 1 [file Data_Sheet_1.docx]
